# Detection of Botulinum Neurotoxin Serotype B at Sub Mouse LD_50_ Levels by a Sandwich Immunoassay and Its Application to Toxin Detection in Milk

**DOI:** 10.1371/journal.pone.0011047

**Published:** 2010-06-10

**Authors:** Miles C. Scotcher, Luisa W. Cheng, Larry H. Stanker

**Affiliations:** Western Regional Research Center, Agricultural Research Service, United States Department of Agriculture, Albany, California, United States of America; University of Wisconsin-Milwaukee, United States of America

## Abstract

**Background:**

Botulinum neurotoxin (BoNT), the causative agent of botulism, a serious neuroparylatic disease, is produced by the anaerobic bacterium *Clostridium botulinum* and consists of a family of seven serotypes (A-H). We previously reported production of high-affinity monoclonal antibodies to BoNT serotype A.

**Methods and Findings:**

Recombinant peptide fragments of the light chain, the transmembrane and receptor-binding domains of the heavy chain of botulinum neurotoxin type B (BoNT/B) were expressed in *Escherichia coli* as GST-fusion proteins and purified. These proteins were used to immunize BALB/cJ mice for the generation of monoclonal antibodies (mAbs). Antibody-producing hybridomas were detected using either a direct binding ELISA binding to plate-immobilized BoNT/B, or with a capture-capture ELISA whereby the capacity of the antibody to capture BoNT/B from solution was tested. A total of five mAbs were selected, two of which bound the toxin light chain and three bound the receptor-binding domain of BoNT/B heavy chain. MAb MCS6-27 was identified via capture-capture ELISA and was the only mAb able to bind BoNT/B in solution under physiological conditions. MAbs F24-1, F26-16, F27-33 and F29-40 were identified via direct binding ELISA, and were able to capture BoNT/B in solution only in the presence of 0.5–0.9 mM sodium dodecyl sulphate (SDS). MAb MCS6-27 and an anti-BoNT/B polyclonal antibody were incorporated into a sandwich ELISA that did not require SDS.

**Conclusions:**

We report here the generation of monoclonal antibodies to serotype B and the subsequent development of a sensitive sandwich immunoassay. This immunoassay has a detection limit of 100 fg BoNT/B, fifty times more sensitive than the mouse bioassay detection limit of 5 pg BoNT/B. Additionally, this assay detected as little as 39 pg/mL of toxin in skim, 2% and whole milk.

## Introduction

Foodborne botulism is a serious condition in which the patient experiences a gradual flaccid paralysis, 18 to 36 hours following consumption of contaminated food. If untreated, botulism can be fatal. Treatment is a lengthy process that may require hospitalization for several months with continuous mechanical ventilation [1]–[2].

Botulinum neurotoxins (BoNTs) are the causative agents of botulism, and are the most potent naturally-occurring toxins known [3]. There are seven serotypes of BoNTs, designated A through G, with serotypes A, B, E and F most frequently associated with human cases of botulism [4]. BoNT/A is the most widely studied and best characterized of the BoNT serotypes - a cursory survey of the scientific literature indicates that there are approximately three times as many publications about BoNT/A than the next most frequent serotype, BoNT/B.

In the United States from 2001 to 2007, a total of 139 cases of foodborne botulism were reported to the Centers for Disease Control and Prevention (CDC). The majority of these cases were caused by intoxication by BoNT/A (76 cases) or BoNT/E (46 cases), with only 10 cases directly linked to consumption of food contaminated with BoNT/B. However, in the same seven years, BoNT/B was the causative agent of 387 of the 663 cases of infant botulism (58.4%) recorded by the CDC [5].

Although BoNT/B is a less frequently observed cause of foodborne botulism, it is nonetheless a significant threat to food safety. The largest recorded outbreaks of foodborne botulism to occur in both the United States and United Kingdom (UK) were attributed to the consumption of food contaminated with BoNT/B. In April 1977 in Michigan, a total of 59 patients were diagnosed with type B botulism, caused by eating a sauce made from improperly home-canned jalapenos. Eleven of the patients required hospitalization, although there were no reported deaths [6]. In June 1989 in the UK, 27 patients were intoxicated (one of whom died) by BoNT/B-contaminated hazelnut yoghurt [7].

At the molecular level, BoNT/A and BoNT/B function in a similar manner. Both toxins are comprised of a 100 kDa heavy chain (Hc) and a 50 kDa light chain (Lc), linked by a single disulphide bond. The Hc functions by binding nerve cells and facilitates the internalization of the Lc, a zinc metalloprotease, into the pre-synaptic neuron at the neuromuscular junction [8]–[9]. The Lc of BoNT/A cleaves synaptosomal-associated protein 25 (SNAP-25) whereas the Lc of BoNT/B cleaves synaptobrevin-2 [10]–[11]. Either cleavage event prevents the docking of acetylcholine-carrying vesicles with the presynaptic membrane, thus blocking the release of the neurotransmitter into the neuromuscular junction and ultimately prohibiting the contraction of the muscle [9].

We recently reported the development of a sensitive sandwich ELISA for the detection of BoNT/A, with a detection limit of 2 pg/mL [12]. The mAbs (F1-2, F1-5 and F1-40) that form the foundation of this sandwich ELISA have been extensively characterized. Binding of these antibodies to the other serotypes of BoNT was undetectable [12]–[14]. Whilst these studies have allowed the development of a test specific for BoNT/A, it is now necessary to develop a novel collection of mAbs to facilitate the development of a sandwich ELISA-based test specific to BoNT/B.

In this article, we describe the production and basic characterization of a collection of monoclonal antibodies specific to BoNT/B. We also report the development of a new sandwich ELISA, capable of detecting BoNT/B in buffer at concentrations undetectable by the mouse bioassay. Finally, we show that this sandwich ELISA can be used to recover BoNT/B from spiked milk samples with minimal sample preparation or modification.

## Materials and Methods

### Recombinant BoNT/B-GST fusion proteins

Commercial enzymes (Phusion High-Fidelity DNA Polymerase, BamHI, XhoI, T4 polynucleotide kinase [3′ phosphatase minus], T4 DNA ligase [New England BioLabs, Inc., Bethesda, MD]) were used according to the manufacturer's recommendation. Primers used were purchased from Integrated DNA Technologies (Coralville, IA) and are shown in Table 1. Plasmid construction and manipulation were performed in *Escherichia coli* TOP10 cells (Invitrogen, Carlsbad, CA) grown aerobically in Luria-Bertani (LB) medium at 37°C supplemented with 100 µg/mL ampicillin [15]. Plasmids or DNA were purified using the QuickClean 5M range of kits (GenScript Corp., Piscataway, NJ). All automated DNA sequencing was performed using the Big Dye Terminator Version 3.1 and XTerminator reagents, and a 3730 DNA Analyzer (Applied Biosystems, Foster City, CA). The 150,000 Da Botulinum neurotoxin type B holotoxin (BoNT/B) produced by *Clostridium botulinum* Strain Okra/Type B1 was used in our experiments (Metabiologics Inc., Madison, WI).

**Table 1:** Primers.

Primer	Sequence	Constructs
B-LcF	GGATCCATGCCAGTTACAATAAATAATTTTAATTATAATG	Lc, L1
B-LcR	CTCGAG **TTA**TTTAACACTTTTACACATTTGTATCTTATATAC	Lc, L2
B-LcintF	GGATCCGCAAGTATATTTAATAGACG	L2
B-LcintR	CTCGAG **TTA**GCCTTTGTTTTCTTGAAC	L1
B-HcF	GGATCCGCTCCAGGAATATGTATTGATGTTG	Hc,H1,H4
B-HcR	CTCGAG **TTA**TTCAGTCCACCCTTCATCTTTAG	Hc, H3, H5
B-HcintR1	CTCGAG **TTA**GCTATTATATTTATTAAACATTTC	H1
B-HcintF2	GGATCCGAAATTTTAAATAATATTATCTTAAATTTAAG	H2, H5
B-HcintR2	CTCGAG **TTA**GCTATATGATTGAATTTTATATC	H2, H4
B-HcintF3	GGATCCGAATATTTAAAAGATTTTTGGGG	H3
M13F	GTAAAACGACGGCCAG	seq. pCR4 plsamids
M13R	CAGGAAACAGCTATGAC	seq. pCR4 plasmids
B-intseqF	CAATAGATAATGCTTTAACTAAAAGAAATG	seq. Hc,H1
B-intseqR	GTGTTCTATCTATATCACCATC	seq. Hc
pGS-F	CAAATTGATAAGTACTTGAAATCC	seq. pGS-21a plasmids
pGS-R	GCTAGTTATTGCTCAGAGG	seq. pGS-21a plasmids

**Figure 1:**
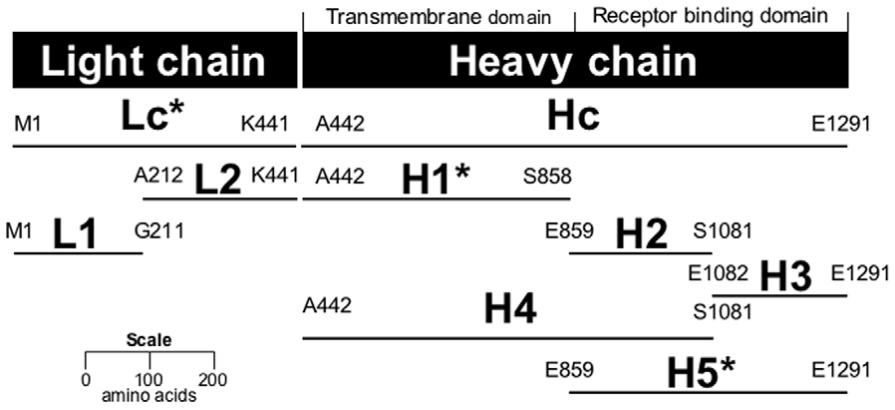
Peptide fragments of BoNT/B light and heavy chains. Diagram is drawn to scale to facilitate size and location comparison between peptide fragments. The transmembrane (A442 to S858) and receptor-binding (E859 to E1291) domains are indicated [27]. Peptide fragments were expressed as fusions to GST at the N-terminal. N- and C-terminal amino acids of each peptide fragment are indicated. Fragments used as antigens to generate monoclonal antibodies in mice are indicated with an asterisk.

Sites for restriction enzymes BamHI (GGATCC), XhoI (CTCGAG), are shown underlined. Stop codons are shown in bold, in the 3′ to 5′ (TTA) orientation. The third column indicates which peptide fragments each primer was used to construct (See Figure 1). Primers used only for sequencing are indicated by the abbreviation “seq.”

Total genomic DNA from *Clostridium botulinum* (Strain Okra/Type B1), generously provided by Eric Johnson (University of Wisconsin, Madison WI), was used as a template to amplify the fragments of the light and heavy chains (Lc, L1, L2, Hc, H1, H2, H3, H4, H5,) using the primers indicated (see Figure 1 and Table 1). Stop codons (TAA) were introduced when not present within the genomic DNA of the cloned region. All subsequent BoNT/B DNA fragments were cloned into plasmid pCR4-TOPO (Invitrogen) to allow sequencing using primers M13F and M13R. Additional primers (B-intseqF, B-intseqR) were required for the longest fragments, Hc and H1. The pCR4-derived plasmids were then digested using BamHI and XhoI, the BoNT/B fragment was purified and ligated into BamHI- and XhoI-digested pGS-21a (Genscript) to yield the correspondently named pGS plasmid (e.g. pGS-H1 for fragment H1). All pGS-21a-derived plasmids were sequenced using primer pGS-F and pGS-R, to confirm the correct integration of the BoNT/A fragment into the vector. The expression and purification of all GST-fusion proteins was performed as previously described [13]. The recombinant DNA methods used in this study were approved by the Institutional Biosafety Committee. DNA sequences determined in this study have been deposied in GenBank and accession numbers ore liste3d in Table 2.

**Table 2:** Characterization of monoclonal antibodies to BoNT/B.

Antibody	Screening method	Hc isotype	Peptides bound*	Epitope location	Accession # of Lc and Hc sequence
F24-1	Direct binding	IgG1	Lc, L2	A212 - K441	Lc – GU799549 Hc – GU799550
F24-4	Direct binding	IgG1	n/d	n/d	n/d
F26-16	Direct binding	IgA	Hc, H3, H5	E1082 - E1291	Lc – GU799551Hc – GU799552
F27-33	Direct binding	IgG1	Lc, L2	A212 - K441	Lc – GU799553Hc – GU799554
F29-38	Direct binding	IgM	n/d	n/d	n/d
F29-40	Direct binding	IgG1	Hc, H3, H5	E1082 - E1291	Lc – GU799555Hc – GU79556
MCS6-27	Capture-capture	IgG1	Hc, H5	E859 - E1291	Lc – GU79557Hc – GU79558

Columns detail the screening procedure used to identify each mAb, the isotype of the Hc of each mAb, the peptides bound, and the epitope location within the holotoxin deduced from the amino acid sequence of the bound peptides. **c**DNA sequences of the variable portion of the Lc and Hc of each mAb can be obtained using the assession numbers indicated.

* = refer to Figure 1 for definition of peptides. n/d = not done.

### Monoclonal Antibody Procedure

The method used for monoclonal antibody production has been described previously [12]. Significant differences from this method are described below.

Solutions of three peptide fragments at the concentrations indicated (Lc, 90 µg/mL; H1, 186 µg/mL; H5, 265 µg/mL) were mixed with Sigma Adjuvant System #S6322 according to manufacturer's instructions (Sigma-Aldrich, St. Louis, MO). Five female BALB/cJ mice (Simonsen Laboratories, Gilroy, CA) were immunized three times at 2-week intervals by intraperitoneal injection (i.p.) of 100 µL of each antigen-adjuvant solution. Two weeks after the third injection, serum was obtained from each mouse and evaluated for anti-BoNT/B antibodies via direct binding ELISA screens. Mice were injected i.p. with 2 µg of the appropriate peptide fragment in 0.01 M phosphate buffered saline (PBS; #P-3813, Sigma-Aldrich) three days prior to being euthanized and cell fusion. Supernatants from cell fusion plates were subjected to screening either by direct binding ELISA or by a capture-capture ELISA screen.

The Institutional Animal Care and Use Committee of the United States Department of Agriculture, Western Regional Research Center approved the experimental procedures used in these studies (protocol #'s 04-1-H-05, 09-3). All animal experiments and husbandry involved in the studies presented in this manuscript were conducted under the guidelines of the U.S. Government Principles for the Utilization and Care of Vertebrate Animals Used in Testing, Research and Training.

### Screening Methods

Direct binding ELISA screens were performed as previously described [12], using microtiter plates coated with 50 µL per well of a 0.1 µg/mL solution of BoNT/B in 0.05 M sodium carbonate buffer, pH 9.6. Binding was visualized using SuperSignal West Dura Extended Duration Substrate (Pierce, Rockford, IL) according to manufacturer's instructions. The plates were incubated for 3 min at room temperature and luminescent counts recorded using a Wallac Victor 2 Multilabel Counter (PerkinElmer Inc., Waltham, MA).

Capture-capture ELISA screens were performed as follows. Unless stated otherwise, 50 µL per well of all solutions were used. Microtiter plates were coated with a 1 µg/mL solution of goat anti-mouse IgG Fc gamma #AP127 (Millipore, Billerica MA) in 0.05 M sodium carbonate buffer, pH 9.6 overnight at 4°C. The IgG solution was aspirated and non-coated sites blocked by adding 300 µL per well of 3% non-fat dry milk in Tris-buffered saline containing 0.05% Tween-20 (NFDM-TBST) and the plates were incubated for 1 h at 37°C. The plates were washed once with 0.05% Tween-20, then cell culture supernatants were added and the plates were incubated at 37°C for 1 h. The plates were washed three times with 0.05% Tween-20, then a solution of BoNT/B in NFDM-TBST (50 ng/mL) was added and the plates were incubated at 37°C for 1 h. Plates were washed three times as before, then a 1 µg/mL solution of anti-BoNT/B rabbit polyclonal antibodies (Metabiologics) in NFDM-TBST was added and the plates were incubated at 37°C for 1 h. Plates were washed three times as before, then a 1 µg/mL solution of goat anti-rabbit HRP-conjugated polyclonal antibodies #A6154 (Sigma-Aldrich) was added and the plates were incubated at 37°C for 1 h. Plates were again washed three times, and binding was visualized as described above.

Cells from the wells giving positive signals for antibody production were cloned by limiting dilution. Hybridomas were then expanded and small amounts (usually less than 10 mL) of ascites fluids obtained (Covance Research Products, Inc., Denver, PA). Antibodies were purified by affinity chromatography on Protein-G (for IgG) or Protein L (for IgA) Sepharose. Bound antibody was eluted with 0.1 M glycine-HCl, pH 2.7 and dialyzed overnight versus PBS. Protein concentrations were determined with a BCA-kit (Pierce) using the microplate method suggested by the manufacturer.

### Antibody Isotyping, Western Blotting, Peptide Binding

All coating, blocking and washing steps of subsequent ELISAs were performed as described for the capture-capture ELISA screen, unless stated otherwise.

The isotype of each antibody was determined using the SBA Clonotyping System/HRP in ELISA format, according to manufacturer's instructions (Southern Biotech, Birmingham AL).

Western Blotting was performed as previously described [12], except that BoNT/B was used instead of BoNT/A. BoNT/B was reduced by the addition of dithiothreitol (DTT) at a final concentration of 10 mM.

Basic characterization of the epitopes of each antibody was performed by direct binding ELISA. The wells of clear microtiter plates were coated with each of the BoNT/B GST-fusion proteins (see Figure 1), incubated overnight at 4°C, blocked with NFDM-TBST and washed. Hybridoma supernatant, diluted 1∶10 in NFDM-TBST, was added to each well and incubated for 1 h at 37°C. The plate was washed, then a 1 µg/mL solution of goat anti-mouse HRP-conjugated polyclonal antibodies #A4416 (Sigma-Aldrich) was added and the plates were incubated at 37°C for 1 h. Plates were again washed three times, then K-Blue substrate (Neogen Corporation, Lexington, KY) was added (100 µL per well) and incubated with agitation for 5 min at room temperature. Stop solution (Neogen) was added (100 µL per well), and absorbance at 650 nm was measured using a VersaMax microplate reader (Molecular Devices, Sunnyvale CA). Each antibody was tested in triplicate.

### Binding of antibodies to BoNT serotypes A through G

Black microtiter plates were coated with the different serotypes of BoNT, A through G (Metabiologics) for direct binding ELISA analysis as described above. Purified anti-BoNT/B monoclonal antibody at a concentration of 10 µg/mL in NFDM-TBST was added to each BoNT serotype, and incubated for 1 h at 37°C. The plates were washed, then a 1 µg/mL solution of goat anti-mouse HRP-conjugated polyclonal antibodies #A4416 (Sigma-Aldrich) was added and the plates were incubated at 37°C for 1 h. Plates were again washed three times, and binding was visualized using SuperSignal West Dura Extended Duration Substrate as described above. Each antibody was assayed against each serotype in triplicate.

### Effects of pH and SDS on capture of BoNT/B from solution by mAbs

Black microtiter plates were coated with the anti-BoNT/B monoclonal antibodies at 10 µg/mL in 0.05 M sodium carbonate buffer, pH 9.6 overnight at 4°C. Non-coated sites were blocked by adding 300 µL per well of 3% non-fat dry milk in Tris buffered saline containing 0.05% Tween-20. Following incubation for 1 h at 37°C, plates were washed three times to remove any residual blocking agent. Solutions of BoNT/B in buffers of various pH and sodium dodecyl sulphate (SDS) concentrations were prepared on a deep-well (2 mL) 96-well plate. Phosphate-buffered saline was added to rows A through G to give a final pH (±0.1) of 5.5, 6.0, 6.5, 7.0, 7.5, 8.0, 8.5 and 9.0, respectively. SDS was added to columns 1 through 12 to give final concentrations of 0, 0.1, 0.2, 0.3, 0.4, 0.5, 0.6, 0.7, 0.8, 0.9, 1.0, 2.0 mM, respectively. The final concentration of BoNT/B was 100 ng/mL in all wells. The deep-well plate was gently agitated to mix the solutions, then 100 µL of each BoNT/B solution was pipetted into the corresponding well on the black microtiter plates. Following incubation for 1 h at 37°C, plates were subsequently treated in an identical manner to the capture-capture ELISA described earlier, using the anti-BoNT/B rabbit polyclonal antibodies, goat anti-rabbit HRP-conjugated polyclonal antibodies and SuperSignal West Dura Extended Duration Substrate to allow detection of BoNT/B capture. Each antibody was assayed in triplicate.

### *In vivo* neutralization of BoNT/B

Random groups of 10 mice (4–5 week old female Swiss Webster mice, 19–21 g; Charles River Laboratories, Portland MI) were injected intravenously (i.v.) into the lateral tail vein with 100 µL of mAbs diluted in PBS to a final dosage of 80 µg mAbs/mouse one hour prior to administration with 100 µL of BoNT/B containing 460 pg (100 mouse iv LD_50_ units) diluted in phosphate gelatin buffer. Control mice were treated with 100 µL of PBS instead of mAbs. Mice were monitored closely over a 14-day period for any symptoms of intoxication, or death. Animal-use protocols were approved by the Animal Care and Use Committee of the USDA, Western Regional Research Center, Albany, CA.

### Cloning and sequencing of monoclonal antibodies

mRNA coding for the anti-BoNT/B monoclonal antibodies was extracted and purified from hybridoma cells as previously described [13]. mRNA was converted to cDNA, and the heavy and light chains of each antibody were amplified by PCR as previously described [16]. The PCR products were gel-purified and treated with polynucleotide kinase (New England BioLabs). Circularized vector pCR2.1 (Invitrogen) was digested with EcoRV and treated with calf intestinal phosphatase (New England BioLabs). The PCR products were ligated into vector pCR2.1, transformed into TOP10 cells, then grown and prepared for DNA sequencing using primers M13F and M13R as described earlier.

### Sandwich ELISA

All combinations of mAbs F24-1, F26-16, F27-33, F29-40, MCS6-27 and the anti-BoNT/B rabbit polyclonal antibody were evaluated as capture and detector pairs for the development of a sandwich assay for BoNT/B detection. Detector mAbs (except the anti-BoNT/B rabbit polyclonal) were biotinylated using the EZ-Link Micro-Sulfo-NHS-Lc Biotinylation Kit (Thermo Scientific, Rockford IL), and then detected using Zymax streptavidin-HRP conjugate (Zymed, San Francisco, CA). The anti-BoNT/B rabbit polyclonal binding was detected using goat anti-rabbit HRP-conjugated polyclonal described earlier (Sigma).

Two pairs of antibodies were identified: F24-1 (capture) and F29-40 (detector); and MCS6-27 (capture) and the anti-BoNT/B rabbit polyclonal (detector). Binding conditions and solutions were optimized (data not shown) to yield the protocols described below. Unless stated otherwise, all solution volumes were 100 µL per well, all incubations were performed for 1 h at 37°C with gentle agitation, and all washes were performed twelve times in water plus 0.05% Tween.

White microtiter plates were coated with mAbs F24-1 or MCS6-27 at a concentration of 5 µg/mL in 0.05 M sodium carbonate buffer, pH 9.6 overnight at 4°C. Non-coated sites were blocked by adding 300 µL per well of 5% non-fat dry milk in Tris buffered saline (pH 8.0) containing 0.05% Tween-20 (NFDM-TBST). Plates were incubated and washed as described above. BoNT/B was added to each plate at concentrations from 5000 to 0 pg/mL in a two-fold dilution series, in TBS pH 6.0 containing 0.6 mM sodium dodecyl sulphate (SDS) for the F24-1-coated plate, or in NFDM-TBST for the MCS6-27-coated plate. Plates were incubated and washed. Biotinylated mAb F29-40 was added to the F24-1-coated plate at a concentration of 5 µg/mL in TBS, pH 8.0 containing 0.6 mM SDS. The anti-BoNT/B rabbit polyclonal detector was diluted 2000-fold in NFDM-TBST and added to the MCS6-27-coated plate. Plates were incubated and washed. Zymax streptavidin-HRP conjugate was diluted 10,000-fold in TBS, pH 8.0 containing 0.6 mM SDS and added to the F24-1-coated plate. Next, goat anti-rabbit HRP-conjugated polyclonal antibody was diluted 2000-fold in NFDM-TBST and added to the MCS6-27-coated plate. Plates were incubated, washed then binding was visualized as described earlier.

### Detection of BoNT/B in milk matrices

The sandwich assay using MCS6-27 (capture) and the anti-BoNT/B rabbit polyclonal (detector) was evaluated for its ability to detect BoNT/B in milk. The sandwich ELISA was performed as described above, with the exception of the capture stage which was performed as follows.

Three milk types (skim, 2% fat and whole) were spiked with BoNT/B at final concentrations of 10000, 5000, 2500, 1250, 625, 312, 156, 78 and 39 pg/mL. Several sample treatments were evaluated in order to defat the samples. Spiked milk samples were centrifuged at 14,000×g for 15 min at 6°C. Other samples were simply diluted in two-fold serial dilutions in blocking buffer [12]. Following defatting or serial dilution, 100 µL of sample was loaded onto the MCS6-27-coated microtiter plate and incubated for 1 h at 37°C with gentle agitation. Recovery of BoNT/B from spiked milk samples is reported as a percentage of the recovery by comparison to a standard curve of BoNT/B spiked into NFDM-TBST. Milk samples were analyzed in triplicate.

## Results

### Characterization of anti-BoNT/B monoclonal antibodies

A total of seven monoclonal antibodies (mAbs) were identified, cloned and characterized (Table 2). Six mAbs (F24-1, F24-4, F26-16, F27-33, F29-38, and F29-40) were identified using a traditional, direct binding ELISA screening method, and a single mAb (MCS6-27) was identified using the capture-capture screen. Isotype analysis revealed that mAb F26-16 was an IgA, F29-38 was an IgM, and the remaining five mAbs were all IgG1s. All of the mAbs possessed kappa light chains.

Sequence analysis of the cloned cDNA coding for the heavy chain variable region and the light chains of each antibody revealed that mAbs F24-1 and F24-4 have identical sequences, and most likely represent independent fusions of a clonally-expanded population of lymphocytes. The remaining five mAbs possess unique variable region sequences for their heavy and light chains, which can be accessed online via the Nucleotide Accession Numbers shown in Table 2. The leader sequences, framework regions, complementarity determining regions (CDRs) and J-regions were identified by inspecting the alignment of the genes for the mAb heavy and light chains to those of other antibody sequences [13]–[14], [17]–[19]. MAbs F24-4 and F29-38 were not used in any further studies.

**Figure 2:**
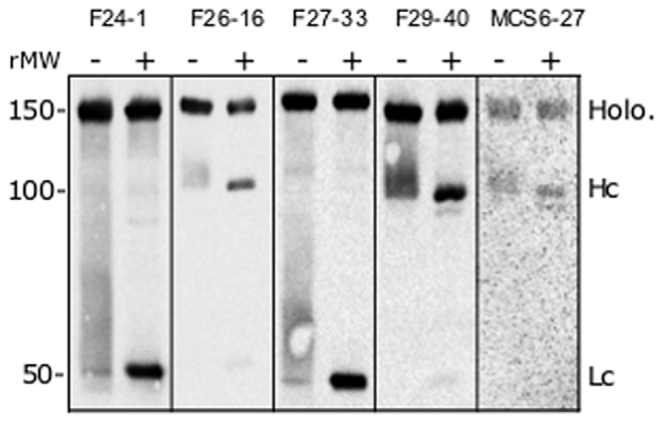
Antibody binding on Western blots of reduced and nonreduced BoNT/B. BoNT/B was separated on 10% SDS-PAGE in the presence (+) and absence (−) of 10 mM DTT, transferred to nitrocellulose membranes and probed with mAbs F24-1, F26-16, F27-33, F29-40 and MCS6-27. Bands showing the intact holotoxin (Holo.), heavy chain (Hc) and light chain (Lc) are indicated. Protein relative molecular weight (rMW) is indicated in kDa.

Each antibody was studied in Western blot experiments, probing reduced and unreduced 150 kDa BoNT/B holotoxin following separation by SDS-PAGE (Figure 2). In this experiment, a constant amount (µg) of each mAb was used to probe the Western blot. Exposure times of Western blots varied; mAbs F24-1, F26-16, F27-33 and F29-40 were exposed for 20 min, whereas the exposure time for MCS6-27 was 120 min. All of the mAbs bound the 150 kDa BoNT/B holotoxin in the unreduced samples. Using reduced samples, mAbs F24-1 and F27-33 bound the BoNT/B Lc, and mAbs F26-16, F29-40 and MCS6-27 bound the BoNT/B Hc. Binding to residual 150 kDa BoNT/B holotoxin was also observed in the reduced samples.

In an effort to more precisely define the binding epitopes for these mAbs, direct binding ELISA experiments were performed to identify which BoNT/B GST-fusion peptides each antibody bound (See Figure 1 and Table 2, columns 4 & 5). MAbs F24-1 and F27-33 were both found to bind the Lc and L2 fragments, but not the L1 fragment or any of the other fragments derived from the BoNT/B heavy chain. The binding epitope for both mAbs is therefore localized to a 230 amino acid fragment of the BoNT/B light chain, between amino acids A212 and K441. MAbs F26-16 and F29-40 bound the Hc, H3 and H5 fragments, but not the Lc, L1, L2, H1, H2 or H4 fragments. The binding epitope for both mAbs is therefore localized to a 210 amino acid fragment of the BoNT/B heavy chain, between amino acids E1082 and E1291. In contrast, mAb MCS6-27 bound fragments Hc and H5, but no binding to any other fragment was detected, indicating that the binding epitope is localized to a 433 amino acid fragment of the BoNT/B heavy chain, between amino acids E859 and E1291. These observations are consistent with the binding data obtained from the Western blot experiments described earlier.

**Figure 3:**
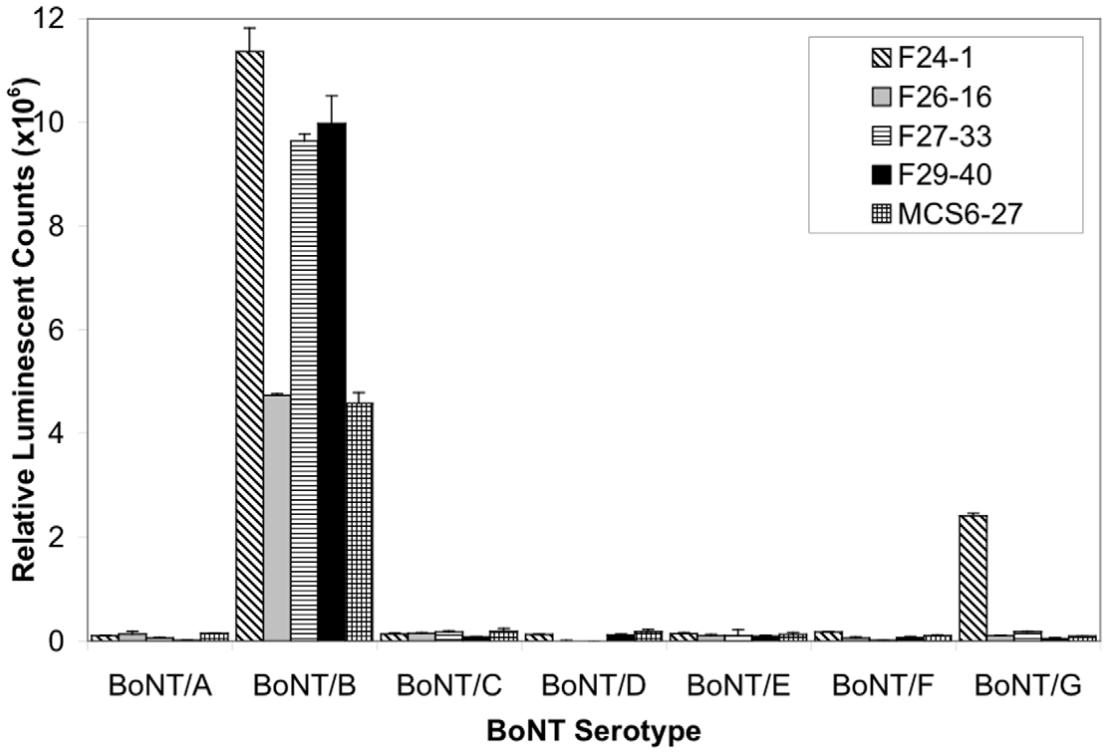
Antibody binding to BoNT serotypes by direct binding ELISA. Antibody binding to black microtiter plates coated with 0.1 µg/mL solutions of BoNT serotypes A through G was measured. Data are presented as average of 3 samples ±1 standard error.

Direct binding ELISAs were performed to test whether each mAb would bind uniquely to BoNT/B, or whether binding to other BoNT serotypes could be detected. These data are summarized in Figure 3. MAbs F26-16, F27-33, F29-40 and MCS6-27 bound only BoNT serotype B, whereas mAb F24-1 bound BoNT serotype B and serotype G.

### Effects of pH and SDS on capture of BoNT/B in solution by mAbs

The ability of each mAb to capture BoNT/B from solution was evaluated by sandwich ELISA. Plates were coated with the capture antibody, BoNT/B was applied (100 ng/mL in 1× TBS), and capture toxin was then detected using a rabbit anti-BoNT/B polyclonal antibody followed by an HRP-conjugated, goat anti-rabbit polyclonal antibody (see methods). Initial experiments indicated that only mAb MCS6-27 was able to capture BoNT/B from solution. BoNT/B was not detected using mAbs F24-1, F26-16, F27-33 or F29-40 as capture antibodies (data not shown). The effects of the pH and SDS concentration of the capture buffer were then evaluated for all 5 mAbs.

**Figure 4:**
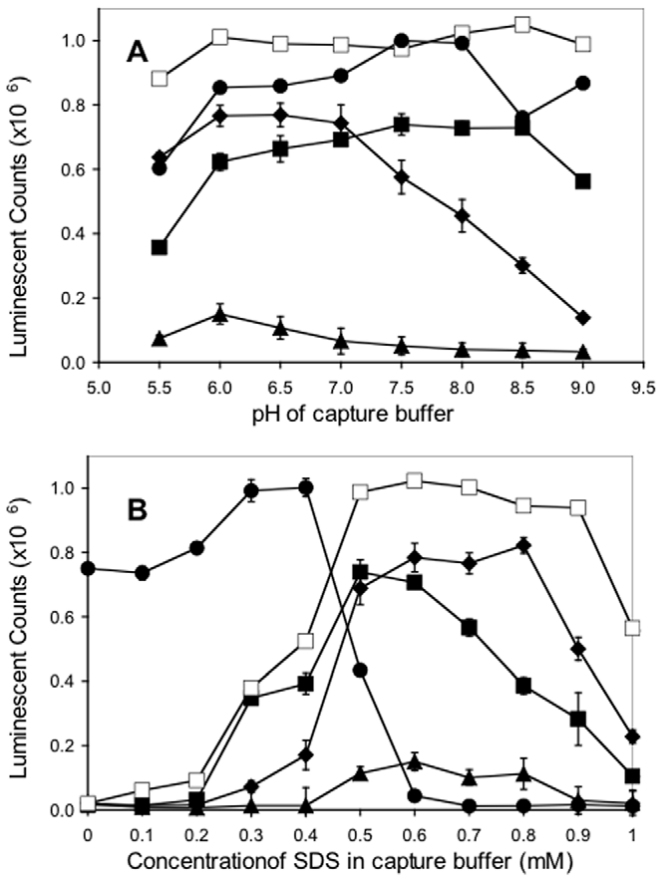
Effects of SDS concentration and pH on BoNT/B capture. Plates were coated with the capture antibody: F24-1 ♦; F26-16 ■; F27-33 ▲; F29-40 □; MCS6-27 ●. **A**. pH of capture buffer (1×TBS) was adjusted for each capture antibody at concentrations of SDS as follows; F24-1, 0.7 mM; F26-16, 0.5 mM; F27-33, 0.6 mM; F29-40, 0.6 mM; MCS6-27, 0.3 mM. **B**. Concentration of SDS in capture buffer was adjusted for each capture antibody at the following pH; F24-1, pH 6.0; F26-16, pH 7.5; F27-33, pH 6.0; F29-40, pH 8.0; MCS6-27, pH 8.0. Data are presented as average of 3 samples ±1 standard error.

The effect of pH, ranging from 5.5 to 9.0, on the ability of the mAbs to capture BoNT/B from solution is summarized in Figure 4A. For the Hc-binding mAbs F26-16, F29-40 and MCS6-27, the pH optimum for BoNT/B capture spanned a broad range from pH 6.0 to 8.5. For the Lc-binding mAbs F24-1 and F27-33, the pH optimum for BoNT/B capture was pH 6.0. It should be noted that in the absence of SDS, no BoNT/B captured by mAbs F24-1, F26-16, F27-33 and F29-40 was detected at any pH tested.

Figure 4B shows the effect of SDS concentration, ranging from 0 to 2.0 mM, on the capture of BoNT/B at the optimal pH for each mAb. MCS6-27 captured BoNT/B at SDS concentrations of 0 to 0.4 mM, reaching an optimal point at 0.4 mM SDS then sharply decreasing such that at SDS concentrations higher than 0.6 mM, BoNT/B capture was not detected. Conversely, no BoNT/B captured by mAbs F24-1, F26-16, F27-33 and F29-40 was detected at 0 mM SDS, but captured BoNT/B was detected with increasing concentration of SDS up to approximately 0.5 mM. Each of these mAbs exhibited the greatest amount of BoNT/B captured at an SDS concentration between 0.5 and 0.8 mM. At SDS concentrations greater than 0.8 mM, the amount of BoNT/B captured declined sharply, with no captured BoNT/B detectable at SDS concentrations of 2 mM (not shown).

### *In vivo* neutralization of BoNT/B

mAbs, F24-1, F26-16, F27-33, F29-40 and MCS6-27 were tested individually for their ability to neutralize BoNT/B holotoxin in a systemic mouse model of intoxication. One hour following intravenous (iv) administration of a mAb against BoNT/B, a lethal dose of BoNT/B (460 pg/mouse or about 100 mouse iv LD_50_) was delivered iv and the animals monitored over time. In the absence of mAbs, intoxicated mice treated with PBS alone died within 3.5–5.5 hrs. Mice pre-treated with 80 µg of F24-1, F26-16, F27-33 or F29-40 were not protected from death and had survival times similar to the PBS treated control mice. In contrast, pre-treatment with 80 µg of MCS6-27 completely protected mice from death as well as any visible symptoms of botulism over the course of 14 days.

### Sandwich ELISA for BoNT/B detection

**Figure 5:**
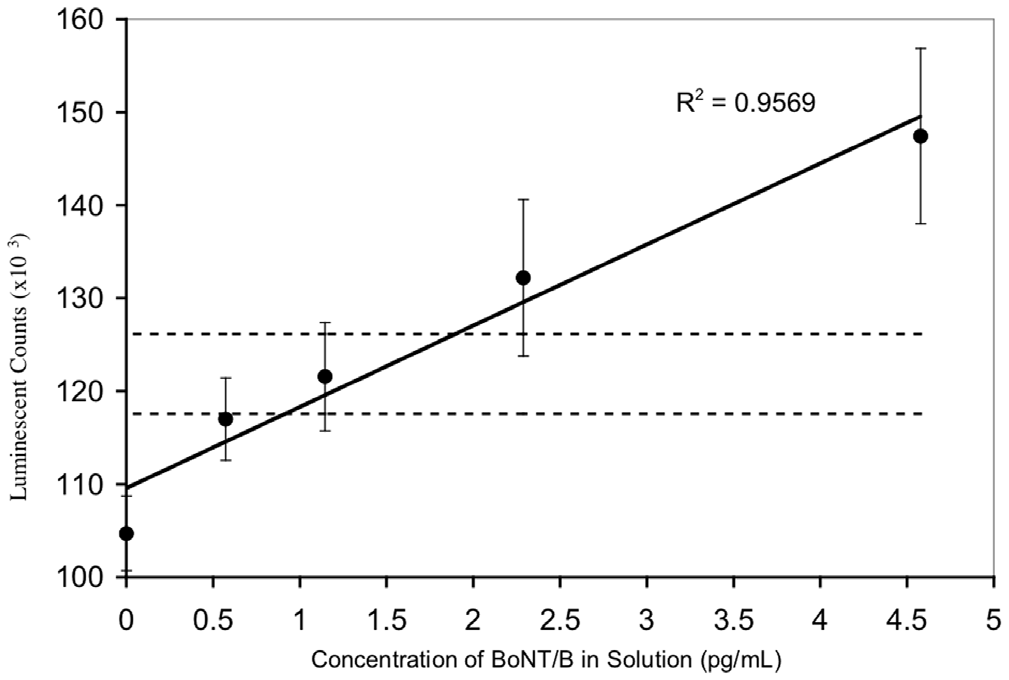
Sandwich ELISA for detection of BoNT/B. MAb MCS6-27 was used as capture antibody, rabbit anti-BoNT/B polyclonal (Metabiologics) was used in conjunction with an HRP-conjugated, goat anti-rabbit polyclonal antibody as detector. Data are presented ±1 standard error, n = 3. Horizontal dashed lines represent the limit of detection (L.O.D., zero toxin plus 3 standard deviations) and the limit of quantitation (L.O.Q., zero toxin plus 5 standard deviations).

All combinations of mAbs F24-1, F26-16, F27-33, F29-40, MCS6-27 and the anti-BoNT/B rabbit polyclonal were evaluated as capture and detector pairs for the development of a sandwich assay for BoNT/B detection. The combination of MCS6-27 (capture) and the rabbit anti-BoNT/B polyclonal (detector) was found to be most sensitive, as shown in Figure 5. This ELISA exhibits a limit of detection (L.O.D., defined as 3 standard deviations above the zero) of approximately 1 pg/mL BoNT/B, and a limit of quantitation (L.O.Q., defined as 5 standard deviations above the zero) of approximately 2 pg/mL BoNT/B. A correlation coefficient (R) value of 0.98 indicates that the regression line is an excellent fit.

### Detection of BoNT/B in milk matrices

The sandwich ELISA described above was used to measure BoNT/B toxin recovery from spiked milk samples (skim, 2% and whole milk). Although several strategies to defat the milk prior to the ELISA were evaluated, it was found that no defatting or dilution process was required for any of the milk types. Recovery of BoNT/B from the spiked milk samples is reported in Table 3. Toxin recovery ranged from 80.4% to 106.7% at BoNT/B spike concentrations from 39 to 20,000 pg/mL.

**Table 3:** Percentage recovery of BoNT/B from milk as determined by sandwich ELISA.

	Spike level (pg/mL)						
	20000	5000	1250	313	156	78	39
Skim milk	98.5±5.7	108.4±4.7	92.3±2.6	100.5±6.9	103.2±3.6	94.1±8.5	101.8±13.7
2% milk	106.2±3.5	102.4±3.7	101.7±2.0	85.7±2.1	106.7±0.3	86.5±1.6	80.4±12.1
Whole milk	100.9±3.7	94.0±2.2	99.2±4.5	97.0±3.6	100.6±5.3	84.9±4.6	82.5±10.3

Data are presented as averages of three independent experiments, ±1 standard error.

## Discussion

We have previously used recombinant GST-fusion peptides of BoNT/A for the successful identification of the epitope sites of mAbs F1-2, F1-5 and F1-40, high affinity mAbs that bind BoNT/A. These studies demonstrated that recombinant peptides of BoNT/A could be successfully employed as surrogates for the BoNT/A holotoxin [13]–[14]. Based upon this observation, we produced three recombinant GST-BoNT/B fusion peptides (Lc, H1, H5) for immunizing mice in order to produce mAbs that bind wild type, intact BoNT/B. It was reasoned that the fusion peptides would not be toxic to the mice, and also would allow the production and identification of mAbs that bind specifically to the light chain (Lc), to the transmembrane domain (H1) and to the receptor-binding domain (H5) of BoNT/B (see Figure 1). This rationale was confirmed by the results reported in this paper.

We identified two mAbs that bound BoNT/B Lc (F24-1 and F27-33) and three mAbs that bound the receptor-binding domain of the Hc (F26-16, F29-40 and MCS6-27). No mAbs that bound the transmembrane domain of Hc were identified. The Hc of mAb F26-16 was found to be an IgA isotype, whereas the Hc of the other four mAbs were IgG isotypes. All five mAbs exhibited kappa light chains.

Binding of each mAb to the collection of BoNT/B GST-fusion peptides shown in Figure 1 was investigated by direct-binding ELISA. Each purified mAb bound the GST-fusion peptide used as an antigen to produce it, but not to any other GST-fusion peptide of BoNT/B. By using smaller GST-fusion peptides, the epitope location for each mAb could be localized to several hundred amino acids.

Both mAbs F24-1 and F27-33 bound the Lc of BoNT/B between residues A212 and K441. MAbs F26-16 and F1-40 bound the receptor-binding domain of the Hc, between E1082 and E1291. The epitope of mAb MCS6-27 could not be further defined because it failed to bind either smaller sub-peptides (H2, H3) of the receptor-binding domain (H5). Since peptides H2 and H3 divide the receptor-binding domain, we speculated that mAb MCS6-27 might bind a region spanning between these two peptides. To test this hypothesis, we constructed a small synthetic peptide 20-mer (EERYKIQSYSEYLKDFWGNP) corresponding to the amino acid sequence spanning peptides H2 and H3. However, no binding to this peptide was observed (data not shown). It is possible that MCS6-27 binds a discontinous conformational epitope that is not entirely present on peptides H2 or H3, or the short synthetic peptide tested. It can only be concluded that the epitope for MCS6-27 lies between amino acids E859 and E1291 of BoNT/B.

The binding of the five mAbs to the BoNT/B holotoxin was confirmed by Western blot analysis. All five mAbs bound the intact BoNT/B holotoxin, with F24-1 and F27-33 selectively binding the Lc of reduced BoNT/B, and F26-16, F29-40 and MCS6-27 binding the Hc of reduced BoNT/B. In order to visualize binding of MCS6-27 to the nitrocellulose-immobilized BoNT/B, the exposure time was increased to two hours, six times greater than that required for the other mAbs. This observation suggests that MCS6-27 binds poorly to the BoNT/B under these conditions, possibly because nitrocellulose-immobilized BoNT/B is in a conformation that is not optimal for the binding of mAb MCS6-27.

All of the mAbs except F24-1 bound only to BoNT/B. In contrast, mAb F24-1 bound to plate-immobilized BoNT/G at approximately 20% the intensity of binding to BoNT/B (Figure 4). A comparison between the BoNT/B Lc between A212 and K441, and the corresponding region of the Lc of BoNT/G [20] revealed 64% identity and 92% similarity in the amino acid sequence, suggesting that some identical or similar residues that comprise the epitope in BoNT/B are also present in BoNT/G.

The mAbs identified via the two different screening strategies displayed markedly different properties. None of the mAbs identified via the traditional direct-binding ELISA were able to capture BoNT/B from solution in the absence of SDS. Conversely, MCS6-27, the only mAb identified via the capture-capture ELISA, was found to be an excellent capture antibody in the absence of SDS. Furthermore, a prophylactic, intravenous (iv) injection of 80 µg of mAb MCS6-27 per mouse protected 100% of the mice studied from death or any symptoms of intoxication from an iv injection of 460 pg (100 mouse iv LD_50_) of BoNT/B, consistent with the observation that mAb MCS6-27 could bind BoNT/B *in vitro* under physiological conditions. The ratio of antibody to LD_50_ units of BoNT/B toxin neutralized was approximately 0.8 µg mAb per 1 LD_50_ unit. A study into the neutralization of BoNT/A in mice using mAb F1-2 revealed a ratio of 0.14 µg F1-2 per 1 LD_50_ unit of BoNT/A [21]. It is possible that a lower quantity of mAb MCS6-27 is sufficient to neutralize 1 LD_50_ unit of BoNT/B, and is the subject of future studies. We hypothesize that since mAbs F24-1, F26-16, F27-33 and F29-40 were unable to bind BoNT/B in physiological buffer, they did not bind toxin *in vivo* and thus failed to protect mice from the neurotoxic effects of BoNT/B. The ability of a mAb to capture antigen from solution appears to be an indicator for toxin neutralization potential. Our data suggest that a capture-capture screen would be more appropriate than a direct binding ELISA if mAbs that neutralize toxin are to be identified.

The observation that all of the mAbs isolated using the direct binding ELISA screen gave strong ELISA titration curves but failed to bind toxin from solution suggested to us that immobilization of toxin on the microtiter plates results in an alteration of some physicochemical properties (e.g., surface charge or tertiary structure) so that a cryptic epitope is exposed that is not available when the toxin is in solution under physiological conditions. If such a hypothesis is correct, mAb MCS 6-27 (which captured toxin in solution and protected from toxin exposure *in vivo*) must bind a surface epitope not altered by immobilization in the wells of the microtiter plate. In an effort to clarify this possibility, we investigated the effects of two factors, the pH and SDS concentration of the capture buffer, on the ability of all five mAbs to capture BoNT/B from solution. The effect of altering the pH was less marked than that of SDS, although both Lc-binding mAbs (F24-1, F27-33) displayed a greater sensitivity to alkaline pH than the other three Hc-binding mAbs (F26-16, F29-40 and MCS6-27). It is possible that the increasingly basic conditions greater than pH 6.0 affected the mAb or the toxin Lc in a manner that caused decreased binding. The same pH conditions did not affect the toxin Hc, or Hc-binding mAbs, in a way that decreased binding. However, in the absence of experiments that assay the structure of both antibody and toxin at each pH, this eventuality cannot be determined.

The concentration of SDS in the capture buffer had a dramatic effect on the ability of the mAbs to bind toxin in solution. SDS is often used at concentrations of 0.1% (∼3.5 mM) in acrylamide gels and associated buffers to bind proteins and cause major conformational changes, commonly known as denaturation. However, there exists a dynamic range of SDS concentration at which SDS binds the protein and induces conformational changes that do not completely denature the protein. At lower concentrations, SDS monomers bind to certain high energy sites on the protein. As the concentration of SDS increases, SDS monomers bind in a cooperative manner ultimately resulting in the saturation of binding. [22]–[23]. It has been shown that the minimum concentration at which SDS can affect protein conformation is 0.1 mM, whereas some proteins can become 100% denatured at SDS concentrations of 1 mM [24]–[25]. Thus, the effects of SDS concentrations up to 1 mM on antibody capture were investigated.

Over the range of SDS concentrations investigated, the capture mAbs separated into two distinct groups, again mirroring the screen by which each mAb was identified. MAb MCS6-27 captured BoNT/B at SDS concentrations between 0 and 0.4 mM, whereas the other four mAbs optimally captured toxin at SDS concentrations between 0.5 and 0.9 mM. A boundary between the two mAb populations fell at 0.4–0.5 mM SDS. We hypothesize that across the range of SDS concentrations from 0 to 1.0 mM, the SDS altered the protein conformation of either BoNT/B or the capture mAb in a manner that facilitated or inhibited binding. It is unlikely that either BoNT/B or MCS6-27 were completely denatured at 0.5 mM SDS, as the concentration of SDS seems too low to have that effect. However it is possible that the conformation of one or both proteins was altered sufficiently, or that key high energy sites on one or both proteins were blocked by monomeric SDS, resulting in the abolition of binding.

More intriguing is how the role of the SDS might relate to the screening methods, specifically to the direct binding ELISA. The surface of the wells on the microtiter plates is described by the manufacturer as “a modified, highly charged polystyrene surface with high affinity to molecules with polar or hydrophilic groups” (Maxisorp, Thermo Fisher Scientific, Rochester, NY). SDS is an anionic molecule, with a ‘tail’ of 12 carbon molecules. It is possible that the SDS and the microtiter plate surface cause similar conformational changes in BoNT/B, thus facilitating the binding of mAbs F24-1, F26-16, F27-33 and F29-40 to BoNT/B in solution or immobilized on the microtiter plate, respectively. We are currently investigating the mechanism by which SDS facilitates binding of these mAbs to BoNT/B in solution.

Our results demonstrate the fundamental importance of using a screening protocol that most closely mimics the format in which the antibody will ultimately be used. Although the capture-capture screen is more time-consuming, costly, and produced fewer antibodies, it is critical for identification of antibodies to be used in sandwich ELISA formats, and possibly for antibodies that can neutralize toxin *in vivo*.

MAbs F24-1, F26-16, F27-33, F29-40, MCS6-27 and the anti-BoNT/B rabbit polyclonal were used in all combinations to identify capture and detector pairs for the development of a sandwich ELISA for BoNT/B detection. Using mAb F24-1 for capture and biotinylated F29-40 for detection, an SDS-dependent sandwich ELISA was constructed. However, the L.O.D. for this assay was approximately 90 pg/mL BoNT/B, almost two orders of magnitude higher than the L.O.D. of 1 pg/mL observed for the most sensitive sandwich ELISA that, under physiological conditions, used mAb MCS6-27 for capture and the anti-BoNT/B rabbit polyclonal for detection. The L.O.Q. was determined to be approximately 2 pg/mL. Since 100 uL of sample is evaluated in the sandwich ELISA described here, the L.O.D. by mass of BoNT/B was 100 fg. The mouse LD_50_ of the BoNT/B preparations used in our laboratory, when injected interperitoneally was found to be 5 pg per mouse (Cheng, unpublished data). Our sandwich ELISA is therefore approximately 50-fold more sensitive than the mouse bioassay for the detection of BoNT/B.

We have previously described the development and application of a sandwich ELISA for the detection of BoNT/A in skim, 2% and whole milk. It was shown that good recoveries were achived when samples were spiked with as little as 312 pg/mL BoNT/A, but that defatting by centrifugation (14,000×g, 15 min, 6°C) or a 10-fold dilution step was necessary to minimize sample interference [12].

The assay described here for the detection of BoNT/B in milk (skim, 2% and whole) does not require any defatting or dilution step. Toxin was readily detectable at a concentration of 39 pg/mL (3.9 pg by mass) in all three milk types, with recoveries of greater than 80%. Although the human oral LD_50_ for BoNT/B has not been determined, the estimated human oral LD_50_ for BoNT/A is 1 µg/kg body weight, or 70 µg for the average-sized human of 70 kg [26]. Assuming a similar toxicity for BoNT/B, the sandwich ELISA can detect as little as ∼1/1,800,000 of the LD_50_.

In summary, we have developed a sensitive assay specific for BoNT/B that is both rapid and easy to use. This assay has the potential to replace the use of the mouse bioassay given its much greater sensitivity of detection. We are currently developing and characterizing additional monoclonal antibodies to BoNT/B to replace the polyclonal detector antibody used here. Our aim is to develop an even more sensitive assay using multiple capture and detector monoclonal antibodies and to explore their application in lateral flow devices.
